# The proper interplay between the expression of *Spo11* splice isoforms and the structure of the pseudoautosomal region promotes XY chromosomes recombination

**DOI:** 10.1007/s00018-023-04912-7

**Published:** 2023-09-08

**Authors:** Teresa Giannattasio, Erika Testa, Monica Faieta, Matteo Lampitto, Daniela Nardozi, Stefano di Cecca, Antonella Russo, Marco Barchi

**Affiliations:** 1 Department of Biomedicine and Prevention, Section of Anatomy, University of Rome Tor Vergata, Rome, Italy; 2 Department of Molecular Medicine, University of Padova, Padova, Italy; 3 Department of Biomedical Science, Lady of Good Counsel University, Tirana, Albania

**Keywords:** SPO11β, SPO11α, PAR, Sex chromosomes, Meiotic recombination, Meiosis, Aneuploidy, Chromosome structure, Splicing, Double strand breaks

## Abstract

XY chromosome missegregation is relatively common in humans and can lead to sterility or the generation of aneuploid spermatozoa. A leading cause of XY missegregation in mammals is the lack of formation of double-strand breaks (DSBs) in the pseudoautosomal region (PAR), a defect that may occur in mice due to faulty expression of *Spo11* splice isoforms. Using a knock-in (ki) mouse that expresses only the single *Spo11β* splice isoform, here we demonstrate that by varying the genetic background of mice, the length of chromatin loops extending from the PAR axis and the XY recombination proficiency varies. In spermatocytes of C57^*Spo11βki/−*^ mice, in which loops are relatively short, recombination/synapsis between XY is fairly normal. In contrast, in cells of C57/129^*Spo11βki/−*^ males where PAR loops are relatively long, formation of DSBs in the PAR (more frequently the Y-PAR) and XY synapsis fails at a high rate, and mice produce sperm with sex-chromosomal aneuploidy. However, if the entire set of *Spo11* splicing isoforms is expressed by a wild type allele in the C57/129 background, XY recombination and synapsis is recovered. By generating a *Spo11αki* mouse model, we prove that concomitant expression of SPO11β and SPO11α isoforms, boosts DSB formation in the PAR. Based on these findings, we propose that SPO11 splice isoforms cooperate functionally in promoting recombination in the PAR, constraining XY asynapsis defects that may arise due to differences in the conformation of the PAR between mouse strains.

**Supplementary Information:**

The online version contains supplementary material available at 10.1007/s00018-023-04912-7.

## Introduction

In eukaryotes, proper segregation of meiotic chromosomes and the production of balanced gametes require recombination between the homologous chromosomes (homologs), a process that is initiated by a programmed wave of double strand breaks (DSBs) introduced by the type IVA topoisomerase-like protein SPO11, along with TOPOVIBL [1–10]. Following formation of DSBs, DNA at the DSBs site is resected, resulting in single-stranded DNA (ssDNA) ends that become the binding site of DNA exchange factors that ultimately leads to the formation of cross-overs (COs) (see [11] and references therein). COs not only shuffle the genome, but also physically link homologs, which ensures they remain associated until segregation occurs at anaphase-I [12, 13]. In males of mouse and humans’ species, recombination between sex chromosomes is more challenging than between autosomes, as DSBs must occur within a short region of homology between them, the pseudoautosomal region (PAR). At least one DSB must form, to allow the generation of the so-called “obligatory CO”, which guarantees proper XY segregation. The haploid mouse genome averages less than one DSB/10Mb, whereas the < 1Mb PAR undergoes one to two DSBs, a frequency that is 10-20-fold higher than the genome average [14]. This indicates that there are mechanisms in place that increase SPO11 activity at the PAR or make it more conductive to the formation of DSBs. In recent years, studies on the mechanisms underlying XY recombination have revealed that proper expression of *Spo11* splice isoforms is key to male sex chromosome recombination. In mammals, *Spo11* has two major splice variants, which are developmentally regulated: Spo11β (44.5 kDa) and Spo11α (40.3 kDa; exon 2 skipped) both including exon 5, the one that encodes the catalytic tyrosine essential for the formation of DSBs [15–17]. By using a mouse transgenic model, it was shown that the expression of the single SPO11β variant causes XY segregation failure and sterility, due to the reduction of the formation of DSBs in the PAR [14]. More recently, it was unexpectedly found that the degree of XY recombination was partially rescued when the transgene was introduced into a different genetic background [18]. This indicates that although germ cells that express only SPO11β are vulnerable to XY recombination-failure, unknown genetic background-dependent factors shape this susceptibility.

The demonstration that the expression of Spo11β does not guarantee recombination at the PAR raised the question of whether, in certain genetic contexts, SPO11α is required to perform this function. In the germ cells, the latter is expressed later than SPO11β, approximately at the time when DSBs are made in the PAR and XY synapse [14], making it a perfect candidate as recombination initiator in the PAR. Nevertheless, no experimental proof of SPO11α role has been provided yet.

The initiation of meiotic recombination requires the expression, along with SPO11 and TOPOVIBL, of auxiliary proteins that are essential for the formation of DSBs in autosomes. In mammals, these include IHO1, MEI1, MEI4 and REC114 [19–24]. XY recombination has additional genetic requirements, demanding expression, and localization on the PAR of ANKRD31, a REC114 binding-protein [25, 26]. Several studies have shown that in yeast and mammals, SPO11-auxiliary proteins (also known as RMMAI proteins [23]) are loaded on the chromosome axis, prior to DSBs formation [19–28]. Nevertheless, according to the yeast model, meiotic DSBs are preferentially localized in the open region of the chromatin, within chromatin loops [29]. This observation has led to the theorization of the “tethering model” which predicts that SPO11 binds to chromatin loops and is successively tethered to the axis, where it is incorporated into the so-called DSB-promoting complex formed by the auxiliary factors [29, 30].

Studies in mice have shown that PAR axes are disproportionately long relative to DNA length (1Mb/mm of axis) compared to autosomes (10-13Mb/mm of axis). Since the density of the loop per millimeter is constant [31], this results in smaller chromatin loops, which according to the tethering model are thought to be more conducive to DSBs [14]. However, whether shorter PAR loops truly boost the formation of DSBs in the PAR is awaiting experimental proof.

By generating a *Spo11β* knock-in hemizygous mouse model (*Spo11βki*/-), we show that in mice with a mixed genetic background (C57BL/6 and 129Sv) the frequency of DSBs formation and recombination in the PAR is highly variable and that a shift to the C57 background greatly reduces such defects. Analysis of PAR ultrastructure revealed that rescue correlates with a shortening of PAR loops and an increased frequency of formation of DSBs. Furthermore, we provide experimental evidence that regardless of PAR structure characteristics, the hemizygous expression of the wild type allele of *Spo11* limits the extent of XY synapsis defects. Finally, by generating *Spo11α* knock-in mice, we prove that SPO11α promotes the formation of DSBs in the PAR, upon concomitant expression of SPO11β.

## Results

### The testes weight of* Spo11βki*/- mice varies with the genetic background

**Fig. 1:**
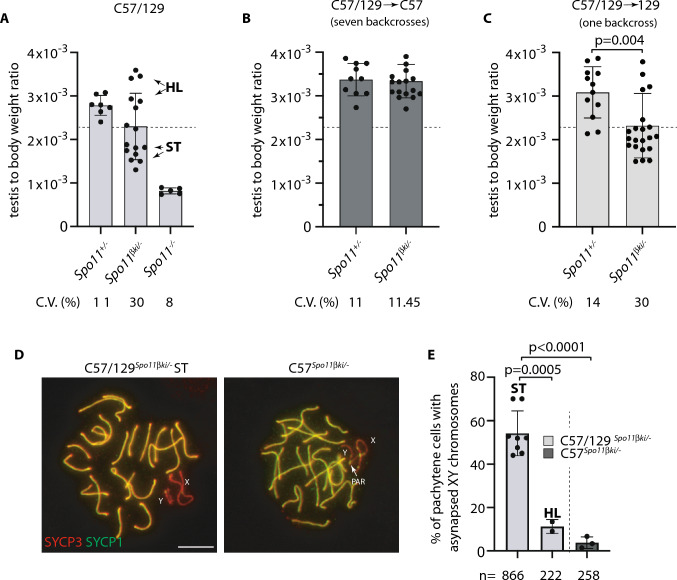
Variability of relative testis weight and XY asynapsis in *Spo11βki*/- mice with a different genetic background. **A** Testis to body weight ratio in mice with the indicated genotypes and genetic background. The dotted line indicates a testis to body weight ratio mean equal to 2.1 × 10^–3^ ± 0.6 × 10^–3^. **B** Testis to body-weight ratio in mice of the indicated genotypes upon seven backcrosses in C57BL/6 background. **C** Testis to body-weight ratio in mice of the indicated genotypes upon one backcross of mice with mixed background in 129/Sv background (HL = heterozygous-like; ST = small testis). In A-C, each dot represents a mouse. C.V. (%) = coefficient of variation. **D** Representative images of spermatocytes stained for the lateral element (SYCP3) and the central element (SYCP1) of the SC. X and Y indicate sex chromosomes; The white arrow points to the PAR. Magnification bar is 10 μm. **E** Frequency of XY asynapsis in nuclei at pachynema. Each dot is a mouse with the indicated genotype; *n* = total number of cells scored for each genotype. The error bars are the mean ± standard deviation (SD) of the mean; *p* = *p* value (two-tailed *t*-test, *p* < 0.05)

In male mammals, death of defective germ cells within the testis, causes an overall reduction in testis weight, so this can be used to quantify spermatogenesis performance (e.g., see [32]).To test how the expression of *Spo11β* affects spermatogenesis when the protein is expressed under normal physiological timing and at allelic dosage, we generated mice expressing a single knock-in allele of *Spo11βb* (thereafter named *Spo11βki/*-) under the control of the *Spo11* promoter (Fig. S1). Mice were created with a mixed (C57BL/6 and 129Sv) genetic background (C57/129^*Spo11βki/−*^), see material and methods and Fig. S2A. Examination of relative testis weight (testis to body-weight ratio) revealed great variability among C57/129^*Spo11βki/−*^ mice, compared to littermates C57/129^*Spo11*+/-^. Indeed, while some C57/129^*Spo11βki/−*^ males had testes with visibly reduced weights, below the mean (i.e., small testis; ST), others appeared indistinguishable from *Spo11*^+/-^ mice (i.e., with a het-like (HL) phenotype) (Fig. 1A). Nevertheless, relative testis weight of ST mice was greater than in *Spo11*^*−/−*^ mice, in which progression of meiosis arrests at zygonema of the first meiotic division [5, 6, 32], indicating that in C57/129^*Spo11βki/−*^ mice the arrest is either incomplete or it occurs beyond zygonema. Given that the mice were of mixed genetic background, we reasoned that the observed phenotypic variability could have been related to background variations. To test this interpretation, we introduced the *Spo11βki* allele into a pure C57/BL6 background (C57^*Spo11βki/−*^ mice) (see Fig. S2B and material and methods); variability was greatly reduced, and testis to body weight ratio turned very similar to *Spo11*^+/-^ (Fig. 1B). Next, to understand whether the phenotype would have worsened in the 129Sv background, we backcrossed C57/129^*Spo11βki/−*^ mice into 129Sv for one generation (see Fig. S2C and material and methods). A single backcross shift was sufficient to worsen the phenotype (compare C57/129^*Spo11βki/−*^ mice in Fig. 1A and Fig. 1C). This was also confirmed by backcrossing C57^*Spo11βki/−*^ mice into 129Sv for one generation (Fig. S2D and S3A). We concluded that in males with the *Spo11βki/-* genotype, the performance of spermatogenesis changes with genetic background.

### Reduced testis to body weight ratio in C57/129^*Spo11βki/−*^ ST mice correlate with failure of sex chromosome synapsis and apoptotic elimination of spermatocytes at metaphase I

In mammals, synapsis of spermatocyte chromosomes occur in the context of the development of a zipper-like proteinaceous structure called synaptonemal complex (SC) [33]. Synapsis begins with the alignment of the homologs at leptonema and is completed by pachynema. Cytologically, cells in leptonema are identified by the appearance of SYCP3 positive stretches of the lateral elements of the SC; progression to zygonema is marked by the assembly of the SYCP1-positive central element of the SC, between pairs of synapsed homologues. At pachynema, autosomes are fully synapsed throughout their entire length and SYCP3 and SYCP1 signals overlap throughout. In contrast, synapsis between XY chromosomes occurs only at the PAR. Thus, a short stretch of SYCP1 forms between chromosomes, only in this region, while the rest of the chromosomes axes is marked by SYCP3.

To probe if variations in testis to body weight ratio in C57/129^*Spo11βki/−*^ mice was related to the proficiency of XY synapsis, we quantified XY asynapsis in our genotypes of interest by staining surface spread chromosomes of C57/129^*Spo11βki/−*^ ST, C57/129^*Spo11βki/−*^ HL and C57^*Spo11βki/−*^ males with anti-SYCP3 and anti-SYCP1 antibodies. While in C57/129^*Spo11βki/−*^ ST, XY synapsis failed in ~ 55% of spermatocytes; the percentage was down to ~ 11% in C57/129^*Spo11βki/−*^ HL and to 4% in C57^*Spo11βki/−*^ mice (Fig. 1D–E), indicating that the reduced testis weight and frequency of XY asynapsis are closely correlated.

In male mice and humans, each seminiferous tubule cross section can be assigned to one of the 12 epithelial stages (numbered I-XII) based on the array of germ cell developmental stages it contains [34–36]. Elimination of MI spermatocytes that have achiasmate homolog pairs (non-exchange) occurs in stage XII by activating the spindle assembly checkpoint (SAC) [14, 37]. To evaluate the occurrence of germ cell loss by apoptosis at stage XII, we combined terminal deoxynucleotidyl transferase dUTP nick end labelling (TUNEL) and anti-H3Ser10 (pH3) staining in testis sections. The latter was used as a marker to identify metaphase I (MI) cells in stage XII. As shown in Fig. S3B and quantified in Fig. S3C, the frequency of MI cell apoptosis was higher in the tubules of C57/129^*Spo11βki/−*^ ST males compared to those of C57/129^*Spo11βki/−*^ HL and C57^*Spo11βki*/−^ males. We concluded that in mice with a *Spo11βki/-* genotype testicular atrophy is related to failure of XY synapsis and apoptotic elimination of defective spermatocytes in stage XII.

### C57/129^*Spo11βki/−*^ ST spermatocytes are defective for the formation of DSBs in the PAR

**Fig. 2:**
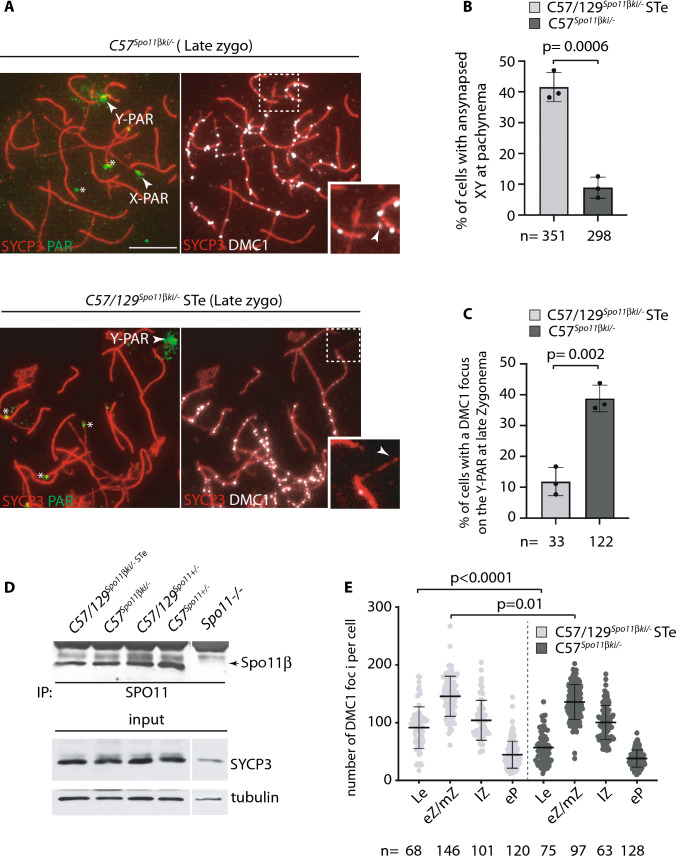
Quantification of the number of DSBs and the expression of SPO11. **A** Representative images of chromosome spreads of late zygotene stage spermatocytes of the indicated genotypes, stained with the anti- SYCP3 and DMC1 antibodies, and hybridized with the PAR FISH probe. Magnified views of the Y chromosomes are shown in the inset. Arrows point to the X-PAR and Y-PAR; *are heterochromatic mo-2 31-bp repeat of either ch4, ch9 or ch13, recognized by the PAR probe. Magnification bar is 10 μm. **B** Quantification of XY asynapsis in juvenile mice with the indicated genotypes and genetic backgrounds. **C** Frequency of the presence of a DMC1 focus on the Y-PAR of cells in A-B. In B and C, each dot is the frequency per mouse; *n* = total number of cells analyzed. **D** Immunoprecipitation (IP) and Western blot analysis of SPO11 expression in testes of mice with the indicated genotypes. *Spo11*^*−/−*^ mice serve as negative controls. Each lane is the expression of one testicle of 4 different mice. The input is a Western blot analysis of the indicated protein markers in total testicular extracts used for IP. SYCP3 and tubulin were normalizers of the number of meiotic germ cells and proteins in the extracts, respectively. **E** Quantification of global DSB numbers in spermatocytes from 12 dpp mice with the indicated genotypes and genetic backgrounds. Each dot indicates the number of DMC1 foci per nucleus. *Le* leptonema; *eZ/mZ* early-mid zygonema; *lZ* late zygonema; *eP* early pachynema. Error bars are mean ± SD; *p* = *p* value (two-tailed *t*-test, *p* < 0.05); *n* = total number of cells scored at each stage (at least three mice per genotype)

To assess whether the defect of XY synapsis in C57/129^*Spo11βki/−*^ ST mice was attributable to the lack of DSBs formation in the PAR, we combined the staining of the SC component SYCP3 and DMC1 (a surrogate marker of DSBs [12, 38, 39]) with that of PAR, using fluorescent in situ hybridization (FISH). The PAR probe recognizes a region at the boundary between the non-PAR region and the PARs of the X and Y chromosomes, and hybridizes with the tandem array of minisatellite mo-2 at the noncentromeric end of chromosomes 4, 9 and 13 [23]. This prevents  iterated unequivocal identification of the X-PAR. On the contrary, the Y-PAR FISH signal has a distinctive pattern, as the FISH staining always extends from the Y chromosome axis to the chromatin loops, forming a distinguishable cloud around the Y-PAR (Fig. 2A). Under physiological conditions, DSB formation occurs with a comparable frequency in both the X-PAR and Y-PAR, mainly at the late zygotene stage, [14]. Therefore, since the Y-PAR is uniquely identified with the PAR FISH probe, we quantified the frequency of DMC1 foci in this region, in late zygonema spermatocytes from C57/129^*Spo11βki/−*^ ST and C57^*Spo11βki/−*^mice. To enrich our samples for germ cells at late zygonema, we prepared chromosome spreads from juvenile (12 dpp) mice. At this time point, apoptosis selection of cells defective in XY synapsis had not yet occurred [32], therefore, the ST phenotype cannot be assessed. To overcome this problem, we evaluated the percentage of XY asynapsis and only included C57/129^*Spo11βki/−*^ mice with at least 35% XY asynapsis in the analysis (henforth referred to as ST equivalent - STe) (Fig. 2B). This value was set according to the correlation between the frequency of XY asynapsis and testis to body weight ratio in adult C57/129^*Spo11βki/−*^ mice (Fig. S3D). Alongside, with this, we analyzed DSBs formation in C57^*Spo11βki/−*^ spermatocytes, in which the average XY asynapsis was less than 10% (Fig. 2B). The analysis of the presence of DMC1 foci in the PAR of late zygotene cells, revealed that the high degree of XY asynapsis correlates with a reduced frequency of the presence of DMC1 foci (Fig. 2C). However, the frequency of DMC1 in the Y-PAR was low compared to the percentage of XY asynapsis. This raised the question of whether DSBs form less frequently in the Y-PAR than in the X-PAR. To test this, we identified both PARs by immunolocalizing ANKRD31, which at the zygotene-pachytene transition and at the early pachytene stages aggregate on PARs (see [26] and below). We analyzed spermatocytes of C57/129^*Spo11βki/−*^ STe (14 dpp) mice with an average XY asynapsis (estimated by SYCP3/SYCP1 staining) equal to 52.5 ± 8%. Of 42 cells with unsynapsed sex chromosomes, 25 (59.5%) had no foci on PARs (Fig. S3E), 10 (23,8%) showed a focus only on the X-PAR, 5 (12%) only in Y-PAR, and 2 (4.7%) in both PARs. The latter are likely cells in which foci are found on both chromosomes, upon release of one DSB from either PARs [14]. We concluded that in C57/129^*Spo11βki/−*^ spermatocytes XY asynapsis occurs as a result of the lack/delayed formation of DSBs on PARs, confirming previous findings [14], and that the frequency of DSBs in the Y-PAR is about twice as low as in the X-PAR.

### Analysis of SPO11 expression in mice with different genetic backgrounds

In mice, the expression of the SPO11 protein below a critical amount may have an effect on DSB levels and chromosome synapsis proficiency [38, 39]. To test whether failure of XY synapsis in C57/129^*Spo11βki/−*^ STe mice was related to faulty expression of SPO11β, we immunoprecipitated it from mouse testis extracts from juvenile mice at 12 dpp. Protein levels among *Spo11*^+/-^ and *Spo11βki/-* mice were comparable (Fig. 2D and Fig. S3F). SPO11 protein levels were also comparable among C57/129^*Spo11*+/-^, C57^*Spo11βki/−*^ and C57/129^*Spo11βki/−*^ HL genotypes, in adults (Fig. S3G**)**. Next, to investigate whether SPO11 function is normal in C57/129 ^*Spo11βki/−*^ STe spermatocytes, we quantified the number of DSBs nucleus wide by co-staining spermatocyte surface chromosome spreads with SYCP3 and DMC1. We did not observe a reduction in DMC1 foci number in C57/129^*Spo11βki/−*^ STe cells compared to C57^*Spo11βki/−*^ spermatocytes. Rather, the average number of foci at leptonema and early mid-zygonema increased slightly in C57/129 ^*Spo11βki/−*^ STe cells (Fig. 2E). We concluded that it is unlikely that the reduced frequency of DSB formation in the PAR of C57/129^*Spo11βki/−*^ STe mice is due to defects of SPO11β expression or function.

### Reduced DSB formation in the PAR of C57/129^*Spo11βki/−*^ STe spermatocytes is not related to defects in the aggregation of the auxiliary proteins of SPO11.

The formation of DSBs in the PAR by SPO11 occurs with the assistance of auxiliary proteins, including IHO1, MEI4, REC114, MEI1 and ANKRD31 (RMMAI complex) [19–26]. Aggregation of RMMAI proteins on the PAR occurs from the preleptotene stage, in advance of the formation of DSBs [23]. To investigate whether SPO11 auxiliary proteins localize normally in PARs of mice with increased XY asynapsis, we monitored the assembly of ANKRD31, MEI4, and REC114 from preleptonema to zygonema in C57/129^*Spo11βki/−*^ STe mice. Spermatocytes from wild type C57 mice were used as a control. To identify their association with the PAR axis, surface chromosome spreads were stained with SYCP3 and the PAR probe. As shown in Fig. S4A-C and quantified in Fig. S4D-F, aggregation of these factors was comparable to that of the control. Furthermore, we immunolocalized aggregates of ANKRD31, MEI4 and REC114 at the zygotene/pachytene transition stage, the sub-stage when most DSBs form in the PAR [14]. To this end, we colocalized them with IHO1, which at this stage forms a blob signal only on X-PAR and Y-PAR [20]. In this case, we never observed cells without ANKRD31, MEI4, or REC114 aggregates in C57/129 ^*Spo11βki/−*^ STe mice (197, 168 and 231 cells analyzed respectively, from three mice per genotype) **(**Fig. S4G**)**. From these results, we ruled out the possibility that a defective aggregation of RMMAI proteins is responsible for the XY asynapsis defects observed in C57/129^*Spo11βki/−*^ STe spermatocytes.

### Spermatocytes from C57/129^*Spo11βki/−*^ STe and C57^*Spo11βki/−*^ mice differ in the high-order chromatin structure of the PAR

**Fig. 3:**
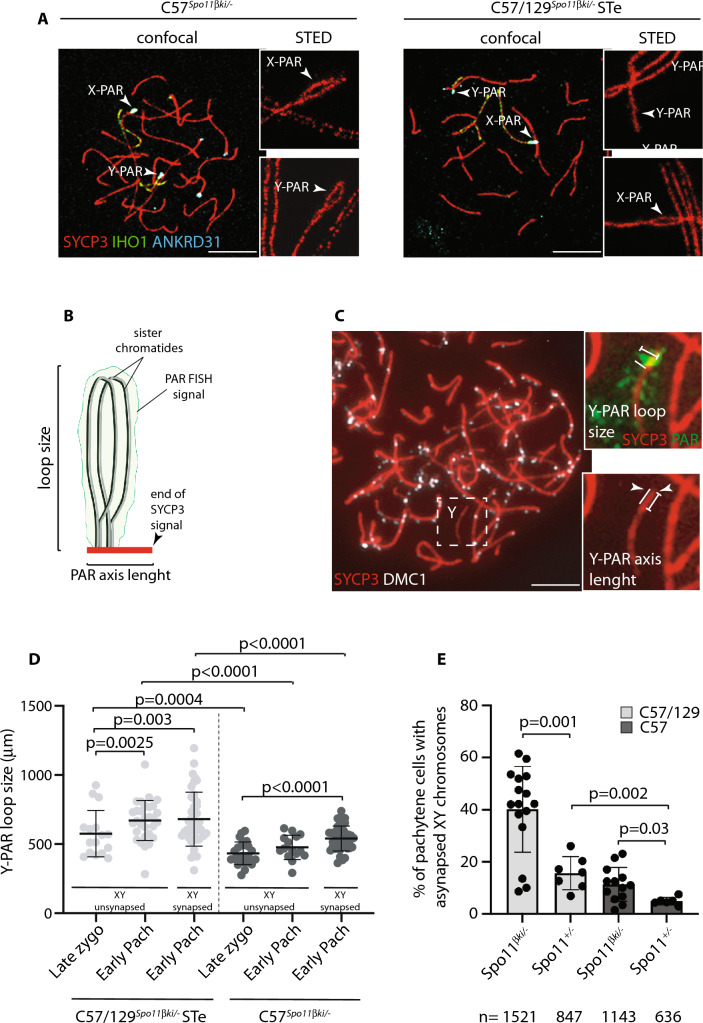
Analysis of the Y-PAR conformation and the proficiency of the XY synapsis. **A** Representative images of cells from 12dpp C57^*Spo11βki/−*^ and ST-equivalent C57/129 ^*Spo11βki/−*^ at late zygotene stage, stained with the indicated markers. IHO1 was used to identify asynapsed chromosomes, while ANKRD31 identifies PARs. Magnifications are STED images of the X and Y PARs. The analysis was performed in two mice per genotype. The numbers of X and Y chromosomes analyzed by STED are as follows: C57 ^*Spo11βki/−*^ mice 21 and 29 respectively; C57/129 ^*Spo11βki/−*^ STe spermatocytes, 26 chromosomes in either case. Arrows point to the X-PAR and Y-PAR. Magnification bar is 10 μm. **B** Schematic of the axis loop structure and the PAR FISH signal. Only one homolog is shown. The length of PAR loops is measured as the orthogonal extension of the FISH signal from the chromosome axis identified by SYCP3. The length of the axis is measured as the maximum distance from the PAR FISH signal to the distal end of the SYCP3-defined axis. **C** Representative image of a late zygotene cell used for the analysis. The dashed square encircles the Y chromosome, identified by the PAR FISH staining pattern (inset). Insets are magnifications showing the Y chromatin extension (green signal, top inset) and the axis extension (red tick signal, bottom inset). The white arrow points to the terminal end of the Y-PAR. In the insets, the white line indicates the length. Magnification bar is 10 μm. **D** Measurements of loop-axis extension from conventional immune-FISH images of cells at late zygonema and early pachynema, in mice with the indicated genotypes. Each dot represents the measurement of a single cell (three mice analyzed per genotype). Pachynema cells with synapsed or asynapsed sex chromosomes were separated into two groups. **E** Frequency of XY asynapsis in nuclei at pachynema, in C57^*Spo11βki/−*^ and C57/129 ^*Spo11βki/−*^ ST mice. Each dot is a mouse; n is the total number of cells scored for each genotype. Error bars are SD, *p* indicates statistical significance (*p* < 0.05), two-tailed *t*-test

In mice, the formation of DSBs in the PAR is preceded by its ultrastructural remodeling that consists of the separation (splitting) by zygonema of the aligned sister chromatid axes, decorated with RMMAI proteins [23]. To monitor potential changes in PAR ultrastructure in C57/129^*Spo11βki/−*^ STe spermatocytes, we analyzed the PAR of surface chromosome spreads of spermatocytes at the zygonema/pachynema transition using Stimulated Emission Depletion (STED) microscopy. To this end, the spermatocyte chromosome axis was stained with anti-SYCP3 antibody, while the sex chromosomes and PARs were identified by IHO1 stain [20]. PARs were also identified by using the anti-ANKRD31 antibody, which forms distinguishable large aggregates on both the X-PAR and Y-PAR [26] (Fig. 3A). By comparing STED images (insets in Fig. 3A), we found that the frequencies of X-PAR axis splitting in late zygonema were comparable between C57/129^*Spo11βki/−*^ STe and C57^*Spo11βki/−*^ mice we used as control (92%, *n* = 34 and 91%, *n* = 33, respectively), while Y-PAR splitting occurred less frequently in C57/129^*Spo11βki/−*^ STe mice (C57/129^*Spo11βki/−*^ ST 84%, *n* = 35; C57^*Spo11βki/−*^ 96%, *n* = 41, *p* = 0.0004 Chi-Square test). Although the physiological role of PAR splitting is still unclear [23], this result suggested a small but noticeable defect in Y-PAR remodeling. In mouse, splitting of the PAR axes are strictly temporally correlated with the remodeling of the PAR chromatin loops and axis. The PARs loops are short at leptonema up to late zygonema, when DSBs are made in the PAR, and lengthen in early to mid-pachynema cells [23]. Correspondingly, the PAR axis is long as soon as it is detectable at leptonema and late zygonema/early pachynema and shortens in the mid-pachytene stage [23]. In our effort to understand the molecular basis of the defect of XY synapsis in C57/129^*Spo11βki/−*^ STe spermatocytes, we sought to study the changes in PAR conformation by measuring the length of the loops and the axis during prophase I in surface spreads of spermatocytes stained with SYCP3 and the PAR FISH probe. We focused on the Y-PAR, as it is uniquely identifiable and its dynamic changes in wild- type cells are well characterized [23]. As a control, we employed C57^*Spo11βki/−*^ males, which are more proficient in XY synapsis (Fig. 2C). The size of loops was defined as the axis-orthogonal extension of the PAR FISH signal, while the length of the PAR axis was determined as the distance from the PAR probe to the end of the SYCP3 axis (Fig. 3B–C) [14, 23, 40]. Comparing cells at late zygonema and early pachynema in C57/129^*Spo11βki/−*^ STe spermatocytes, the average size of PAR loops at late zygonema was shorter than in early pachynema, confirming previous results [23]. This was true regardless of whether the XY synapses had just occurred at early pachynema (Fig. 3D). Similarly, the Y-PAR loops of C57^*Spo11βki/−*^ spermatocytes at late zygonema were shorter compared to cells at early-pachynema with synapsed sex chromosomes. An upward trend in average loops length was also observed in early pachytene-stage cells with asynapsed XY, although the difference did not reach statistical significance. Remarkably, the comparison of FISH signals among cells of C57/129^*Spo11βki/−*^ STe and C57^*Spo11βki/−*^ mice indicated that the PAR loops of C57^*Spo11βki/−*^ mice were constitutively more compact than those of C57/129^*Spo11βki/−*^ STe cells (Fig. 3D), consistent with smaller loops. Side-by-side analysis of the length of the Y-PAR axis showed that it shortened slightly in early pachynema cells of C57^*Spo11βki/−*^ mice, while no significant variations were found in C57/129^*Spo11βki/−*^ STe cells (Fig. S5A). The latter was expected, as the shortening of the PAR axis is generally measurable by mid-pachynema [23]. We did not find mid-pachytene cells at the 12 dpp time point; therefore, shortening of the PAR axis at this more advanced stage could not be tested. From these experiments, we concluded that the spermatocytes of C57/129^*Spo11βki/−*^ STe and C57^*Spo11βki/−*^ mice differ for the high-order chromatin structure of the PAR.

### Interplay between PAR ultrastructure and expression of the *Spo11* wild type allele

In mice carrying, a wild type allele of *Spo11* in the mixed background (C57/129^*Spo11+/-*^), relative weight of the testes is high and less variable compared with that of C57/129^*Spo11βki/−*^ mice (Figs. 1A, C and S3A). To investigate how these phenotypes correlate with the frequency of XY asynapsis, we quantified it in the genetic models of our interest. As shown in Fig. 3E, sex chromosome asynapsis was less frequent in C57/129 ^*Spo11*+/-^ mice compared to C57/129^*Spo11βki/−*^ males. This indicates that the expression of the full set of *Spo11* splice-isoforms by the wild type allele promotes XY recombination and synapsis better than the *Spo11βki* allele. The subsequent comparison of XY asynapsis in C57/129^*Spo11*+/-^ and C57^*Spo11*+/-^ males pointed out that the latter are the most proficient. To test whether this correlated with a shortening of the PAR loops length, we measured it in juvenile C57/129^*Spo11*+/-^ and C57^*Spo11*+/-^ mice. PAR loop length in spermatocytes with a C57 background were significantly shorter (Fig. S5B), confirming our previous results (Fig. 3D). Shortening of PAR loops also correlated with a recovery of XY asynapsis in cells from C57^*Spo11βki/−*^ males (Fig. 3E). We concluded that reduced length of the PAR loops in the C57 background and the expression of a wild type set of *Spo11* splice-isoforms, both impacts on XY recombination, likely by distinct mechanisms, in cooperation with each other.

### The function of *Spo11β* on PAR is boosted by the concomitant expression of *Spo11*α

**Fig. 4:**
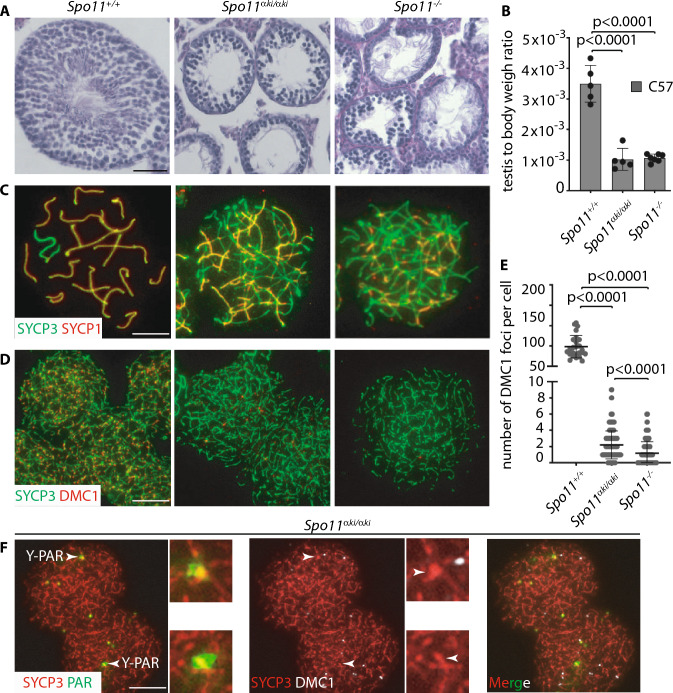
Characterization of C57^*Spo11αki/αki*^ mice phenotype. **A** Histological analysis of testes from mice of the indicated genotypes; hematoxylin and periodic acid shift staining of testis sections from adult mice. Round spermatids and sperm are apparent in the wild type testes. In contrast, tubules in C57^*Spo11αki/αki*^ and *Spo11*^*−/−*^ mice, lacks haploid cells. Two to three mice were analyzed for each genotype. Magnification bar is 50μm. **B** Relative Testis to body weight ratio of mice with the indicated genotypes. **C** Representative images of surface-spread spermatocyte nuclei stained with antibodies recognizing SYCP3 and SYCP1. **D** Surface-spread spermatocytes stained with antibodies that recognize SYCP3 and DMC1. **E** Quantification of DMC1 foci in mice with the indicated genotypes; we analyzed three mice per genotype. Each dot on the graph represents a single cell. The error bars are SD, p indicates statistical significance (*p* < 0.05), two-tailed *t*-test. **F** Surface spread spermatocytes from mice of the indicated genotype, stained with antibodies recognizing SYCP3, DMC1 and with the PAR FISH probe. In C-D and F magnification bars are 10μm

**Fig. 5:**
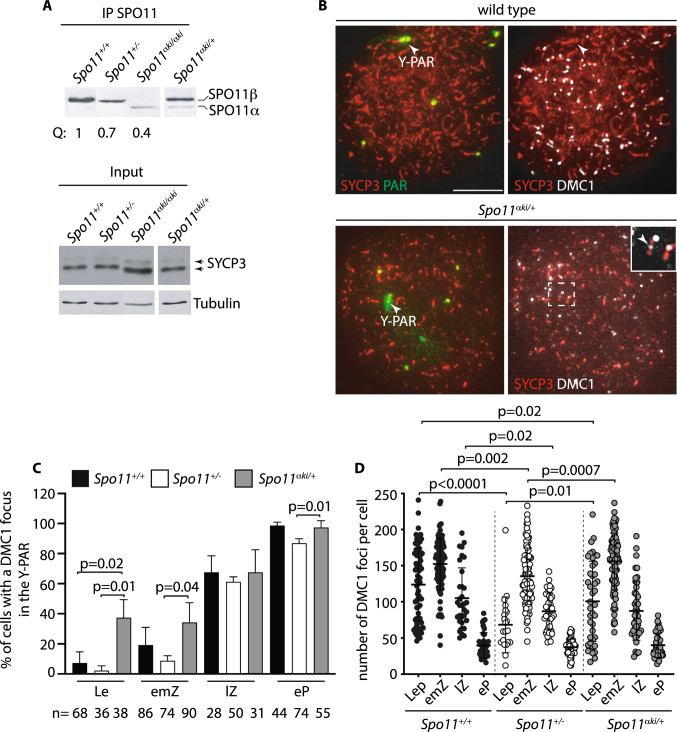
Expression of SPO11and phenotypic characterization of the C57^*Spo11α/*+^ mice phenotype. **A** IP Western blot analysis of SPO11 expression in mice of the indicated genotypes. Each lane is the expression of one testis of four mice. Input is Western blot analysis of the indicated protein markers in total testicular extracts. SYCP3 and tubulin in input were normalizers of the amount of meiotic germ cells and proteins in the extracts, respectively. **B** Surface spread spermatocytes from mice of the indicated genotypes, stained with antibodies that recognize SYCP3, DMC1 and with the PAR FISH probe. Y-PAR was identified by the staining pattern of the PAR FISH probe. The inset is a magnification of the Y chromosome. The white arrows point to the PAR. Bar is 10 μm. **C** Quantification of the number of DMC1 foci in the Y-PAR at different substages of spermatogenesis, in mice with the indicated genotypes (three mice per genotype); *n* = number of cells analyzed per stage. The error bars are SD, *p* indicates statistical significance (*p* < 0.05); one tailed t-test. **D** Quantification of the global number of DMC1 foci, in spermatocytes of mice with the indicated genotypes. The error bars are SD, *p* indicates statistical significance (*p* < 0.05), two-tailed *t*-test

SPO11α conserves the catalytically active tyrosine residue of *Spo11* required for its DSB formation activity [15, 16]; therefore, it is a potentially catalytically active isoform. With the goal of testing the ability of this isoform to form DSBs, we generated a knock-in mouse model that expresses it under the control of the *Spo11* promoter (Fig. S1). Mice homozygous for the *Spo11*α*ki* allele were generated on a C57 background (C57^*Spo11αki/αki*^). Analysis of the morphology and relative testicular weight of these mice revealed that they phenocopied *Spo11*^*−/−*^ mice [5, 6] (Fig. 4 A, B). Furthermore, histological observation of the ovaries of adult mice revealed that females were also phenotypically similar to *Spo11*^*−/−*^ [5, 6], as primordial follicles could not be observed in the cortex (Fig. S5C). Consistent with these observations, staining of spermatocyte spread chromosomes with SYCP3 and SYCP1 antibodies, revealed that, just as *Spo11*^*−/−*^ spermatocytes [5, 6], C57^*Spo11αki/αki*^ cells were not able to progress beyond a zygotene-like stage (Fig. 4C). Successive quantification of the number of DSBs in spermatocytes using DMC1 as a surrogate marker, showed that the number of DSBs was extremely low in C57^*Spo11αki/αki*^ cells compared to wild type mice, although slightly higher than in *Spo11*^*−/−*^ spermatocytes (Fig. 4D, E). To confirm this result, we also quantified the number of γH2AX patches, which mark DSB sites regardless of the DMC1 assembly [41]. Again, numbers of γH2AX patches were slightly increased compared to *Spo11*^*−/−*^ mice (Fig. S5D-E). Confirming the failure of proper formation of DSBs, the histological analyses of C57^*Spo11αki/αki*^ testes revealed that, as previously demonstrated in *Spo11*^*−/−*^ mice [5, 32], spermatocytes underwent massive cell death (Fig. S5F). Next, we went one step further by testing whether one of the few DSBs that form in C57^*Spo11αki/αki*^ spermatocytes occur in the PAR. To this end, we immunolocalized DMC1 in the PAR of surface chromosome spreads of C57^*Spo11αki/αki*^ cells in combination with SYCP3 and the PAR FISH probe (Fig. 4F). Of the three mice analyzed, we never observed DMC1 foci in the Y-PAR of cells in leptonema (*n* = 53) and found foci in 6/376 nuclei in the zygonema-like stage (1.2% ± 0.3). Conversely, DMC1 foci were never found in the Y-PAR of *Spo11*^*−/−*^ cells at any stage (*n* = 218 cells, from three mice). We concluded that in C57^*Spo11αki/αki*^ males, DSBs form with extremely low efficiency on both non-sex and sex chromosomes. To investigate whether such a phenotype was traceable to a low level of the protein, we immunoprecipitated SPO11 from C57 wild type, C57^*Spo11*+/-^ and C57^*Spo11αki/αki*^ testes. Samples were collected from 12 dpp mice to compare testes with similar progression of meiosis. SPO11α expression in C57^*Spo11αki/αki*^ mice was visibly reduced compared to SPO11β in wild type and C57^*Spo11*±^ spermatocytes (Fig. 5A). This suggests that the low frequency of DMC1 foci in C57^*Spo11αki/αki*^ spermatocytes is at least in part attributable to the low protein level.

Considering that under physiological conditions, SPO11α is expressed in prophase I, later than SPO11β, [6, 15, 42, 43], we speculated that another reason why the proficiency of DSB formation in the PAR and autosomes of C57^*Spo11αki/αki*^ spermatocytes is low is because it lacks SPO11β. As shown in Fig. 5A (left panel), in our *Spo11*α knock-in model, the protein is expressed with an early timing compared to wild type, as it is already well detected in testes of 12 dpp mice, when in wild type mice is only observed SPO11β. Taking advantage of this characteristic, we generated mice expressing one wild type allele of *Spo11* in combination with the *Spo11αki* allele (i.e., C57^*Spo11*α*ki/*+^ mice). After verifying the expression of both splice isoforms (Fig. 5A, right panel), we quantified the number of DSBs in the PAR, comparing it with C57 wild type and C57^*Spo11*+/-^ spermatocytes, which by this age only express SPO11β. Our prediction was that if the function of SPO11β in the PAR is enhanced by concomitant expression of SPO11α, DSBs should form with greater efficiency in the PAR of C57^*Spo11*α*ki/*+^ cells at leptonema and early zygonema compared to cells from control genotypes. This expectation was met. Quantification of DMC1 foci in the Y-PAR of leptotene stage cells revealed that the frequency of DSBs was increased by five folds in C57^*Spo11*α*ki/*+^ spermatocytes compared to wild type C57 cells and by over 16 folds compared to cells from C57^*Spo11*+/-^ mice. A smaller increase was also observed in the early/mid zygotene and early pachytene stages, compared to C57^*Spo11*+/-^ cells (3.9 and 1.1, respectively) (Fig. 5B, C). From this observation, we concluded that SPO11β function in the PAR is augmented by the concomitant expression of SPO11α. Interestingly, quantification of DMC1 foci on whole chromatin of C57^*Spo11ki*α*/*+^ spermatocytes at leptonema and early/mid zygonema revealed that “global” DSBs increased less (1.4 and 1.1 folds, respectively) than in the PAR (Fig. 5D). This indicates that the expression of SPO11α is mainly functionally related to recombination initiation in the PAR.

### C57/129^*Spo11βki/−*^ ST mice are prone to sex chromosome aneuploidy in sperm

Previous studies have shown that in male mice prone to sex chromosome asynapsis, fertility and differentiation of aneuploid sperm are functions of the degree of XY asynapsis [44]. If XY pairing fails in not more than ∼50% of sperm, activation of the spindle assembly checkpoint (SAC) does not have an obvious impact on sperm production and mice are fertile [44]. Consistent with the fact that in C57/129^*Spo11βki/−*^ ST mice XY synapsis fails in ~ 55% of cells, mice had reduced but still abundant spermatozoa in the cauda of the epididymis (Fig. S6A) and were fertile (Table S1). Next, to understand whether such mice generated sperm aneuploid for the sex chromosomes, we subjected cells collected from the cauda of the epididymis to FISH with probes against the X and Y chromosomes. A fluorescent in situ hybridization probe for chromosome 8 served as an internal control for correct identification of aneuploid sperm vs diploid ones (Fig. S6B). In C57/129^*Spo11βki/−*^ ST mice, the percentage of sperm nuclei containing both X and Y or no sex chromosomes was increased (Fig. S6C). We conclude that C57/129^*Spo11βki/−*^ ST males are prone to formation of aneuploid gametes.

## Discussion

### Proficiency of XY recombination changes with mouse strain and correlates with variations of the ultrastructure of the PAR

Previous studies demonstrated that the expression of the single *Spo11β* splice-isoform in mouse predisposes defective XY recombination and synapsis [14]. However, the degree of XY recombination failure varies by mouse strain [18]. It remained unknown whether this occurred because a lack of concurrent expression of SPO11α, an altered recruitment of the RMMAI factors, alterations in the high-order chromatin structure of the PAR, or by other mechanisms. Herein, by generating a *Spo11β* knock-in model that expresses the protein under the control of its own promoter, we show that in mice with mixed genetic backgrounds (C57BL/6 and 129Sv), formation of DSBs in the PAR and XY synapsis is impaired with high variable frequency. On the contrary, the introduction of the knock-in allele in a pure C57 background greatly restored SPO11β function at the PAR and XY synapsis, providing a comparative model to investigate the mechanisms shaping the proficiency in PAR DSB formation. Comparison of SPO11β expression in C57/129^*Spo11βki/−*^ ST and C57^*Spo11βki/−*^ males revealed equal levels of protein expression and bulk of DSBs. This excluded any potential detrimental effect on XY recombination due to variable expression of the ki allele in different genetic backgrounds. Subsequent analysis of the presence and timing of aggregation of RMMAI factors in the PAR also ruled out the possibility of their defective recruitment as causative for XY recombination failure. In wild type, concomitantly with RMMAI proteins aggregation, the PAR undergoes notable ultrastructural rearrangements prior to DSB formation. These include the separation of the aligned sister chromatids from each other (splitting), the elongation of the PAR axis, and the shortening of the chromatin loops. These changes have been proposed to be essential for the recombination, pairing, and segregation of XY chromosomes [23]. However, whether alterations of the PAR ultrastructure correlate with XY recombination defects has never been experimentally tested. By analyzing PAR splitting in C57^*Spo11βki/−*^ and C57/129^*Spo11βki/−*^ ST spermatocytes, we observed in the latter, a small difference in the frequency of Y-PAR splitting, this suggested a defect in remodeling of the Y-PAR, which correlated with a more pronounced reduction of DSBs in the Y-PAR than in the X-PAR. To date, the functional significance of splitting of the PAR is unclear. Two strongly related hypotheses have been proposed. One is that separated axes would accommodate a considerable amount of SPO11 RMMAI proteins required for sufficient DSBs [23]. Alternatively, splitting could prevent unnecessary ineffective inter-sister recombination, to support repair of DSB by homologous recombination [45]. Given that in our model we did not observe substantial defects in hyperaccumulation of RMMAI proteins on the PAR, we favor the latter hypothesis. Next, deepening the analysis of the ultrastructure of the PAR, we analyzed loop/axis remodeling. We focused on the Y-PAR and found that in C57/129^*Spo11βki/−*^ ST spermatocytes loops are considerably longer than those of C57^*Spo11βki/−*^ cells. This is in line with the model that envisions short loops being more conducive to the formation of DSBs [14, 23]. We concluded that defective XY recombination in C57/129^*Spo11βki/−*^ ST cells is likely due to the ultrastructural conformation of the PAR.

### Interply between *Spo11* splicing isoforms expression and PAR conformation

Comparison of XY recombination proficiency in mice expressing a single wild type *Spo11* allele with that of mice with the *Spo11βki/-* genotype revealed that the former were more proficient in XY synapsis. This indicated that one wild type allele of *Spo11* is superior to the *Spo11βki* allele, in promoting XY recombination. On the other hand, *Spo11*^+/-^ with a C57 background were the most proficient in sex chromosome synapsis among *Spo11*^+/-^ mice. This was also correlated with the presence of shorter PAR loops. We concluded that strain-dependent changes in PAR ultrastructure and the expression of a wild type *Spo11* allele cooperate in promoting XY recombination, likely by different mechanisms. Based on these results we hypothesized that, in cases where the ultrastructure of the PAR is unfavorable for the formation of DSBs (i.e., long loops; for instance, for a constitutive 3D organization of PAR chromatin), the concomitant expression of other splicing isoforms of *Spo11* additional to SPO11β may compensate for such characteristic. The Y-PAR is the one receiving DSBs with lowest frequency in C57/129^*Spo11βki/−*^ mice with defective XY synapsis. Therefore, it is likely the one that benefits most from the expression of additional *Spo11* splicing forms.

### The concomitant expression of SPO11β with SPO11α promotes DSB formation in the Y-PAR

The major alternative splice isoform of *Spo11* expressed in addition to *Spo11β* in *Spo11*^+/-^ mice, is Spo11α. Thus, we took a step forward by generating a new knock-in model that expresses SPO11α under the control of *Spo11* promoter. Phenotypic characterization of C57^*Spo11*α*ki/*α*ki*^ mice showed that although proficiency of DSB formation in bulk chromatin and PAR was extremely low compared to normal mice, it was above the level in *Spo11*^*−/−*^ cells. That said, SPO11α expression was surprisingly low in our model, reduced by approximately 2.5 folds of the level of SPO11β in age-matched *Spo11*^+/-^ mice. The reason for this attenuated expression is unknown. It could be due to reduced expression of the knock-in allele. However, as the backbone construct is identical to that of the β isoform, it is unlikely. Therefore, we favor the hypothesis that the mRNA or the protein of the α isoform is less stable. Regardless of the underlying mechanism, this observation suggested that the inefficient formation of DSBs by SPO11α in our model might be linked to its reduced expression. However, comparison with a mouse model in which SPO11β is reduced at an apparently comparable level [38], indicated that in these cells the number of DMC1 foci was approximately 50-fold higher than in C57^*Spo11*α*ki/*α*ki*^cells (~ 100 DMC1 foci on average in early mid zygonema in Tg(*Spo11β*) ± vs. 2.2 on average in zygotene-like cells of C57^*Spo11*α*ki/*α*ki*^ mice). This indicates that while SPO11α conserves the catalytic domain [15, 16] it has low DSB activity. Consistent with this interpretation, it has been shown that generation of DSBs by SPO11β requires its heterotetramerization with two TopoVIB-like (TOPOVIBL) subunits to adopt the structure required for DNA cleavage [10, 46]. TOPOVIBL is apparently unable to physically interact with SPO11α [10], which likely represents a limit in the activity of SPO11α. Given that SPO11α molecules can self-interact [43], we speculate that in C57^*Spo11*α*ki/*α*ki*^ mice, protein complexes containing αα dimers form, and have no (or a very reduced) function in the formation of DSBs in autosomes and low DSB formation efficiency in the PAR. In wild type cells, the formation of DSBs in the PAR occurs at a time point when both SPO11β and SPO11α are expressed [14]. Therefore, it was predicted that the formation of DSBs in the PAR could be favored by their concomitant expression. To test this interpretation, we took advantage of the fact that SPO11α is expressed earlier than wild type in our knock-in mouse model, with the same timing as that of SPO11β. We asked if DSBs form with a greater efficiency in leptonema and early zygonema cells of juveniles *Spo11*^*αki/*+^ mice than in wild type and *Spo11*^+/-^ controls. Beside the low level of SPO11α expression, the percentage of cells with a DMC1 focus on the Y-PAR at leptonema was increased considerably compared to *Spo11*^+/-^ cells; an increase was also observed in cells at early mid-zygonema and early pachynema. This demonstrates that SPO11β function in the PAR is boosted by concomitant expression of SPO11α. Remarkably, the enhancement of DSB generation by this mechanism occurs in animals with a C57 genetic background, indicating that the implementation in DSB formation in the PAR due to splice isoforms co-expression is in addition to the presence of a favorable PAR ultrastructure. Successively, quantification of nucleus wide DSBs in *Spo11*^*αki/*+^ cells at leptonema and early-mid zygonema, revealed the DSBs increased only modestly, compared to the increased frequency of DSBs in the PAR. This underlines the functional specificity of SPO11α for XY chromosomes recombination. In a recent study it was demonstrated that a direct interaction of TOPOVIBL with REC114 is required in males for the formation of DSBs in the subtelomeric regions and at PAR and that the binding of REC114 to TOPOVIBL is mutually exclusive with ANKRD31 [47]. Given that *Ankrd31* is essential for the formation of DSBs in the PAR [25, 26], we envision the possibility that the protein complex that leads to DSB formation at the PAR might involve the interaction of SPO11β with TOPOVIBL and REC114 and that of SPO11α with ANKRD31. The latter would possibly be mediated by a (TOPOVI type B-like) protein, which is perhaps expressed with the same timing of SPO11α and preferentially or exclusively binds to ANKRD31. Alternatively, SPO11α interacting with both ANKRD31 and REC114 could serve as an intermediary in the interaction of the SPO11β/TOPOVIBL heterotetramer with the PAR. In this regard, we recently demonstrated that SPO11α co-immunoprecipitates with REC114 in vivo [48], indicating that when this short form of SPO11 is expressed, it interacts with pre-DSB promoting factors, likely promoting DSB activity at the PAR. More studies will be needed to clarify how SPO11 splice isoforms interact dynamically with TOPOVIBL and/or additional type B-like proteins, as well as with RMMAI proteins while cells progress through prophase I.

### Defective recombination initiation between XY chromosomes leads to differentiation of aneuploid sperms

One important output of our study is that alterations in the frequency of XY recombination initiation and synapsis closely correlates with the differentiation of XY aneuploid spermatozoa. Therefore, in the long term, understanding of the XY recombination mechanisms at the molecular level has the potential to illuminate the genetic origin of paternally-derived cases of Klinefelter syndrome (47, XXY) [49, 50] and male infertility when this is associated with high levels of XY aneuploidy [51].

## Materials and methods

### Targeting of *Spo11* cDNA

cDNAs of Spo11β-bclI (Spo11βb) or Spo11α-bclI (Spo11αb) splice isoforms and a downstream pA sequence (from the SV40 TpA of pcDNA3.1 vector) were synthetized by Gene art (Thermo Fisher). Each cassette was then cloned into a pPGK-Keo vector, containing the kanamycin/neomycin resistance cassette (Keo), flanked by two lox-P (L) sites, downstream a hybrid intron (HI) structure. After retrieval of genomic DNA (BAC clone #RP23-20N4) into pDTA vector, the HI-cDNA-pA-LKL cassette was inserted into the genome, with deletion of the entire exon 1, to obtain the pDTA Spo11βb and pDTA Spo11αb vectors (Fig. S1A). Following linearization with AsiSI (New England Biolabs), DNA was electroporated in A9 ES cells (129 Sv and C57BL/6N background); mouse core facility, EMBL, Rome. Targeted cells (TA) were identified by southern blotting using the 5’ probe, following AflII (New England Biolabs) digestion and injected into 8 cell-stage C57BL/6N embryos. To remove the LKL cassette, the founder males carrying the TA allele were crossed with Deleter-CRE mice (C57BL/6N background) to obtain mice carrying either the *Spo11βb Ki* or *Spo11αb Ki* alleles (*Spo11βki/* + or *Spo11αki/* +). Cassette removal was verified by Southern blotting, after digestion of genomic DNA with the Afl II restriction enzyme and hybridization with the 5 'probe' (Fig. S1B).

### Generation of *Spo11βki* and *Spo11αki* mice models

C57/129^*Spo11βki/−*^ and C57/129^*Spo11*+/-^ matching controls were obtained by mating C57/129^*Spo11βki/*+^ founders with *Spo11*^+/-^ mice with a mixed (C57BL/6 and 129 Sv) background [5] (Fig. S2A). C57^*Spo11βki/−*^ mice and C57^*Spo11*+/-^ matching controls were obtained by first crossing C57/129^*Spo11βki/*+^ mice with wild type C57BL/6 for seven generations. Next C57^*Spo11βki/*+^ were mated with C57^*Spo11*+/-^ mice (Fig. S6B). The latter were obtained from C57/129^*Spo11*+/-^ mice after seven backcrosses in the C57BL/6 background. The backcross of C57/129^*Spo11βki/−*^ mice into 129 (one backcross), was achieved by first crossing C57/129^*Spo11βki/−*^ females with wild type 129 males. Next, C57/129^*Spo11βki/*+^ and C57/129^*Spo11*±^ of the F1 were mated with each other (Fig. S2C). C57^*Spo11αki/ αki*^ mice were obtained by backcrossing C57/129^*Spo11αki/*+^ founders into C57 for seven generations. Then, C57^*Spo11αki/*+^ males and females were mated with each other. The phenotype of C57^*Spo11αki/ αki*^ males was compared with that of C57^*Spo11−/−*^, obtained by mating mice with C57^*Spo11*+/-^ genotype. C57^*Spo11αki/*+^ and C57^*Spo11*+*/*+^ controls were obtained by mating C57^*Spo11αki/*+^ mice. In all cases, to minimize variability from strain background, mice were compared with controls from the same litter or from the same mating involving closely related parents. Each analysis has been made for at least a minimum of 3 animals per genotype.

### Genotyping

Genotyping was performed by conventional PCR, using 2X MyTaq Red Mix (Bioline Aurogene, BIO-25044) of tail tip DNA. Primer pairs (Integrated DNA Technologies, IDT) are indicated in supplementary table 2.

### Morphometric analysis of the testes

Testis was collected from 45 to 60 dpp old mice. Each animal was euthanized and weighted; testes were removed and weighed as well. The mean between testis weight was calculated and normalized to body weight to minimize the difference in testis size due to mouse physiology.

### Histology and immunostaining of tissues sections

The testes and ovaries were collected and fixed overnight (ON) at 4° C in 4% paraformaldehyde (PFA) or Bouin fixatives (Sigma, HT10132). The fixed samples were embedded in paraffin (Thermo SCIENTIFIC Histoplast, 6774006). Sections of 5 μm were stained with periodic acid–Schiff (PAS) (Schiff’s fuchsin sulfite reagent, Sigma, S5133) and with hematoxylin (VWR, 340374 T). Images were captured using a Zeiss Axioskop bright-field microscope equipped with a color CCD camera.

### Terminal deoxynucleotidyl transferase dUTP nick end labelling (TUNEL) of testis sections

After deparaffinization and rehydration, sections were treated to unmask the antigenic epitope, using Tris–EDTA citrate buffer, pH 7.8 (UCS Diagnostic, TECH199) for 30 min in steam and subjected to the TUNEL assay, according to the manufacturer’s instructions, using the Roche In Situ Cell Death Detection Kit (POD) (cat. N. 11684817910). To identify stage XII, testis sections were co-stained with anti- pH3 antibody (see Table S3). For each genotype, we analyzed stages XII from at least three testis sections per mouse, cut at 50–80 μM distance from each other. The number of stages XII analyzed for each genotype are as follow: C57/129^*Spo11βki/−*^ ST = 48; C57^*Spo11βki/−*^ = 13; C57/129^*Spo11βki/−*^ HL = 40.

### Preparation of spermatocyte chromosome spreads, immunostaining, FISH hybridization and analysis of DMC1 foci in the PAR

The spermatocyte surface chromosome spreads were prepared and stained according to [40]. Primary and secondary antibodies used are listed in Supplementary Tables 3 and 4. Hybridization of the PAR by FISH was performed as previously described [40], labeling the X chromosome probe BAC RP24-500I4 which in mice strains under study hybridizes at the PAR boundary (~ 10.5 kb overlap with the X-PAR and Y-PAR) and extends into the non-homologous part of the X. The X and Y PARs were scored as positive for a DMC1 focus when the following criteria were fulfilled: the DMC1 focus co-localized with the SYCP3 signal and localized to the stretch of SYCP3 staining corresponding to the PAR, identified either by FISH or co-staining with ANKRD31. Images were captured using a Leica CTR6000 digital inverted microscope connected to a charge-coupled device camera and analyzed using the Leica software LAS-AF (Leica) for fluorescent microscopy. Super-resolution analysis was performed using the STEDYCON confocal microscope (Abberior Instruments).

### Isolation of sperm and XY FISH

Spermatozoa have been collected from the cauda epididymis as described in [52]. Samples were stored at – 80° C. To perform FISH spermatozoa smears were obtained, fixed through washes in ethanol series, then 10 min in Methanol (Sigma, 32213)/Acetic Acid (VWR 20104.298) (3:1) on ice. Preparations were incubated in 10 mM DTT, 0.1 M Tris–HCl (pH 8.00) for 30 min on ice and air-dried. Mouse X, Y probes (MCEN-XY-10-GRRE, Empire Genomics) probes and chromosome 8 probe (BAC clone RP23126A1, BACPAC Genomics, CA USA) were mixed in the hybridization buffer provided with the Empire Genomics kit. Sex chromosome probes were labelled respectively with green and red fluorescent fluorophores, while the autosomal probe was either colabelled with Alexa Fluor-488 and Alexa Fluor-594 dUTP or labelled with Alexafluor-647 dUTP (Molecular Probes, Invitrogen), following a nick translation assay. Hybridization was performed accordingly to Empire Genomics instructions. Slides were mounted with antifade solution (Vectashield; Vector Laboratories, Newark, CA, USA) containing 1 μg/mL of 4′- 6- diamidino- 2- phenylindole (DAPI).

Slides were analyzed under a motorized fluorescence microscope (Zeiss Axio Imager.M1) equipped with a monochromatic CCD camera (Photometrix, Coolsnap HQ2). Analyses were carried out under a 100X oil immersion objective (N.A. = 1.30). For capture and image analysis the MetaMorph software (7.1.3.0, Molecular Device) and the MetaVue software (7.8.11.0, Molecular device) were respectively used.

### Immunoprecipitation of SPO11 and Western blot analysis

Immunoprecipitation and Western blot have been performed according to [48, 53]. Briefly, testes from adult or juvenile mice were decapsulated and lysed using the Pierce IP Lysis Buffer (Thermo Fisher Scientific, 87787) complemented with proteases inhibitors 2X (Roche, cOmplete Tablets EDTA-free, 04693132001), phosphatases inhibitors 1X (Sigma-Aldrich, Phosphatase Inhibitor Cocktail 3, P0044) and benzonase (ChemCruz, sc-202391A) according to manufacturer instructions. Supernatants were incubated with Dynabeads Protein-A (Thermo Fisher Scientific, 1002D) loaded with the mouse monoclonal anti-SPO11-180 antibody (table S3) which recognizes specifically both SPO11β and SPO11α isoforms [14], in rotation at 4 °C. Mouse anti-IgG2A (table S3) served as a control. At the end of incubation, the dynabeads were washed three times with Lysis buffer and eluted with standard Laemmli buffer. The samples were fractionated on 8–12% SDS-PAGE and transferred to a PVDF membrane (GE Healthcare, Amersham Hybond P Western blotting membranes, GE10600023) using a semi-dry transfer system (Hoefer, TE22). For Western Blot (WB) analysis, membranes were probed with primary antibodies diluted in BSA 5%/TBS 0.1% Tween 20 (TBS-T). Secondary antibodies were diluted in 5% nonfat dry milk (AppliChem, A0830)/TBS-T. The primary and secondary antibodies used are indicated in supplementary tables S3 and S4. WB signals were detected using ECL reagent (BIO-RAD, Clarity Western ECL Substrate, #170–5061). Quantification of SPO11 protein level was performed by densitometry using ImageJ software. Values were normalized against SYCP3/tubulin or REC8/tubulin ratio in total extracts. SYCP3 or REC8 were used as markers for spermatocytes content in the testis.

### Analysis of PAR ultrastructure

PAR loops and axis lengths were measured according to the method described by Acquaviva et al. [23].

### Statistical analysis

Statistical analysis was performed using GraphPad Prism 9 for Macintosh (GraphPad Software, San Diego, CA). Data were expressed as mean ± SD or mean ± SEM, as detailed in the figure captions.

### Artwork

The artwork was created with Adobe Photoshop and Illustrator 2022.

### Supplementary Information

Below is the link to the electronic supplementary material.

Supplementary file1 (PDF 26 KB)

Supplementary file2 (PDF 85 KB)

Supplementary file3 (PDF 200 KB)

Supplementary file4 (PDF 630 KB)

Supplementary file5 (PDF 298 KB)

Supplementary file6 (PDF 7402 KB)

Supplementary file7 (DOCX 32 KB)

## Data Availability

The authors confirm that the data supporting the findings of this study are available in the article and its supplementary materials.
