# Supplementary figures and images for: The proper interplay between the expression of Spo11 splice isoforms and the structure of the pseudoautosomal region promotes XY chromosomes recombination
Source: Cell Mol Life Sci. 2023 Sep 8;80(10):279. doi: 10.1007/s00018-023-04912-7 (PMC10491539; doi:10.1007/s00018-023-04912-7)

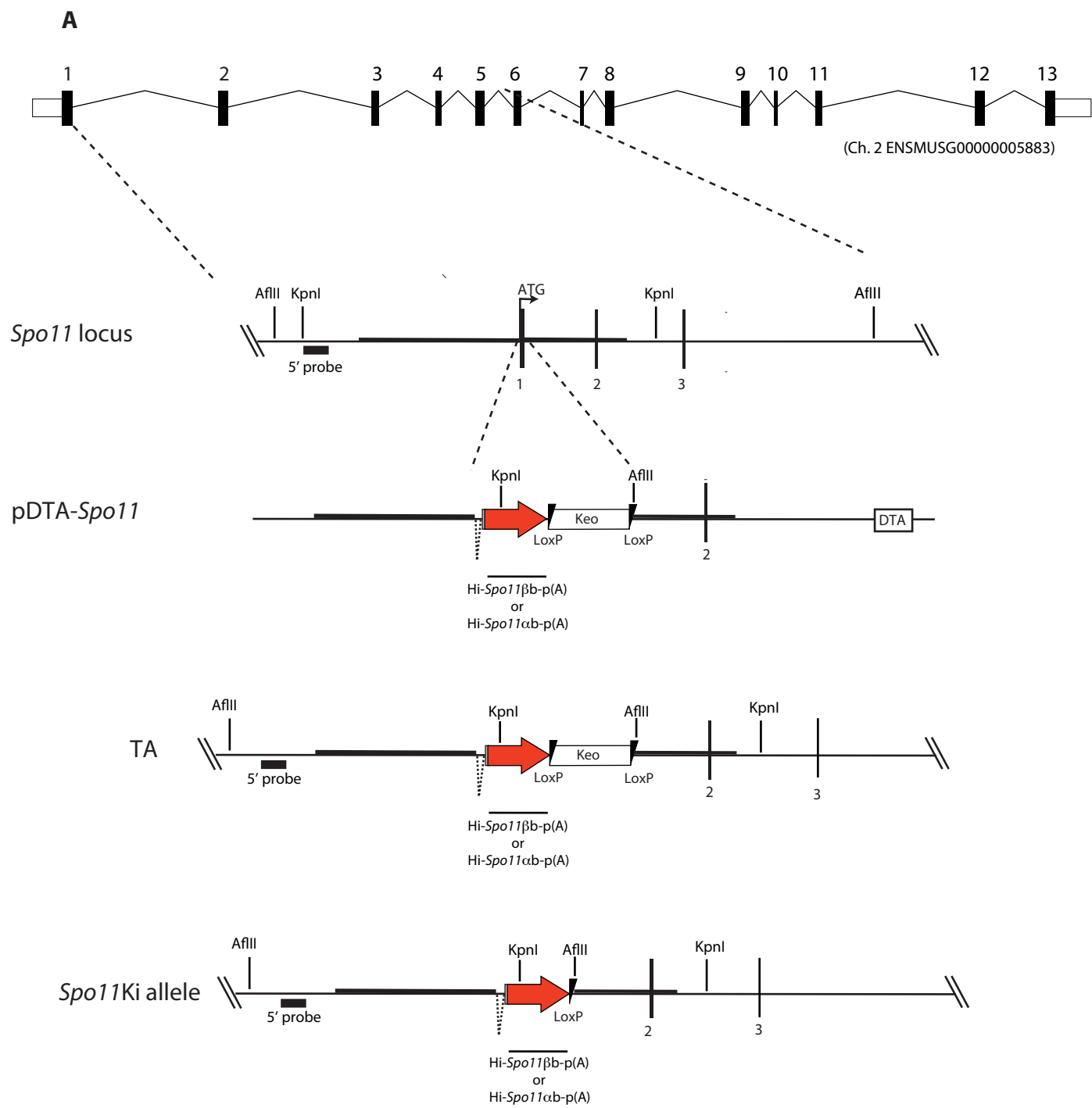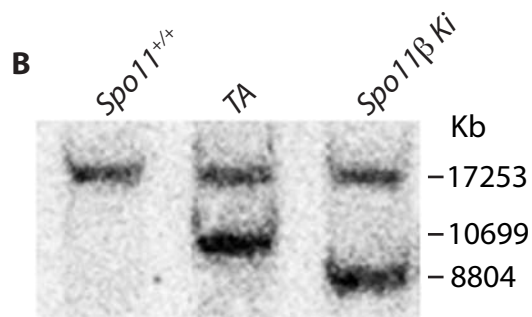

Fig. S1

Supplement: Supplementary file 1 — Supplementary file1 (PDF 26 KB) [file 18_2023_4912_MOESM1_ESM.pdf]

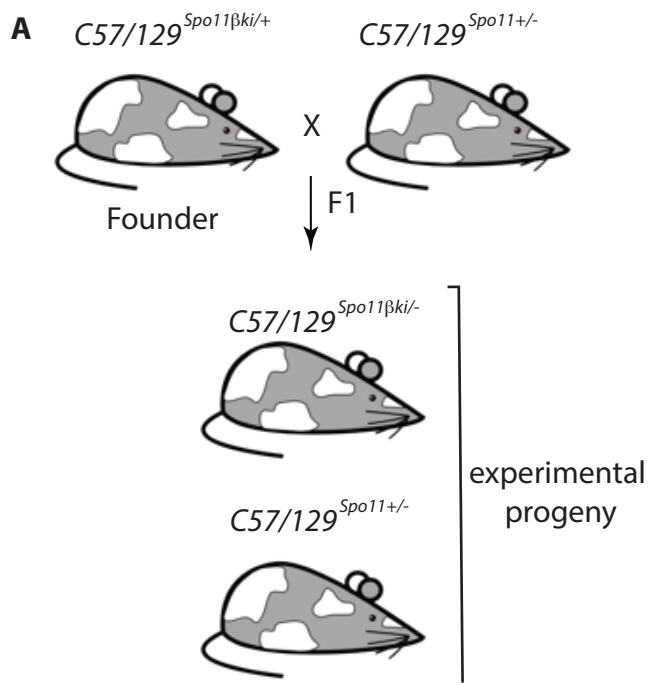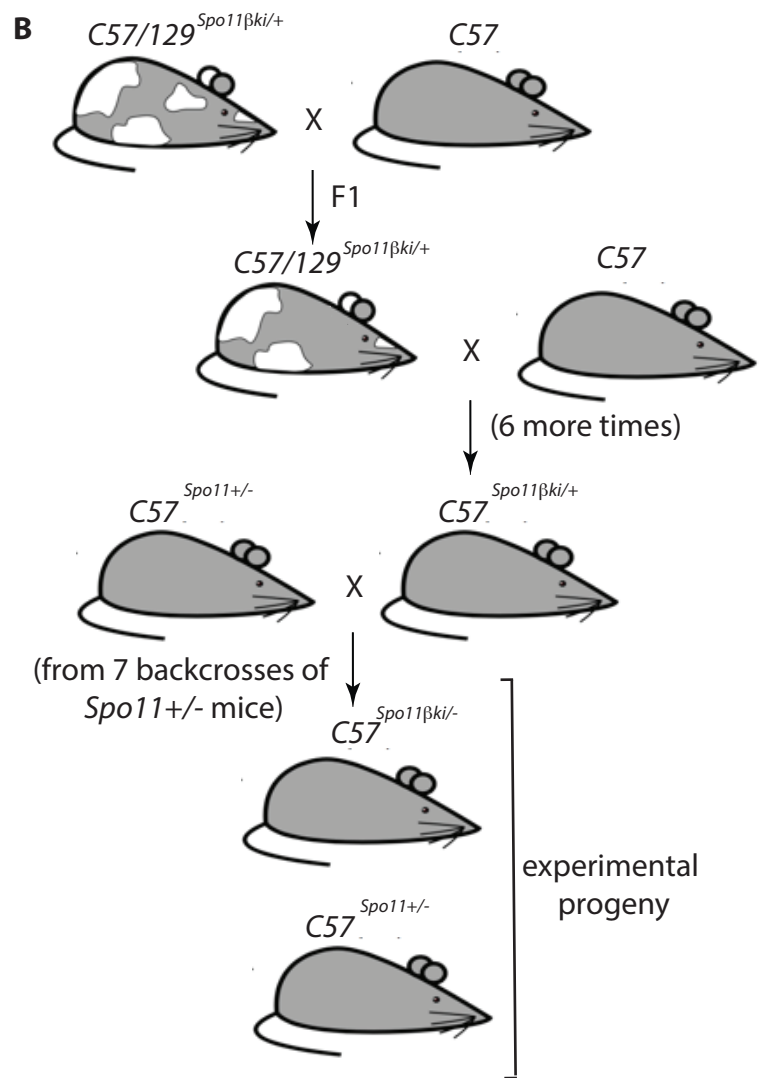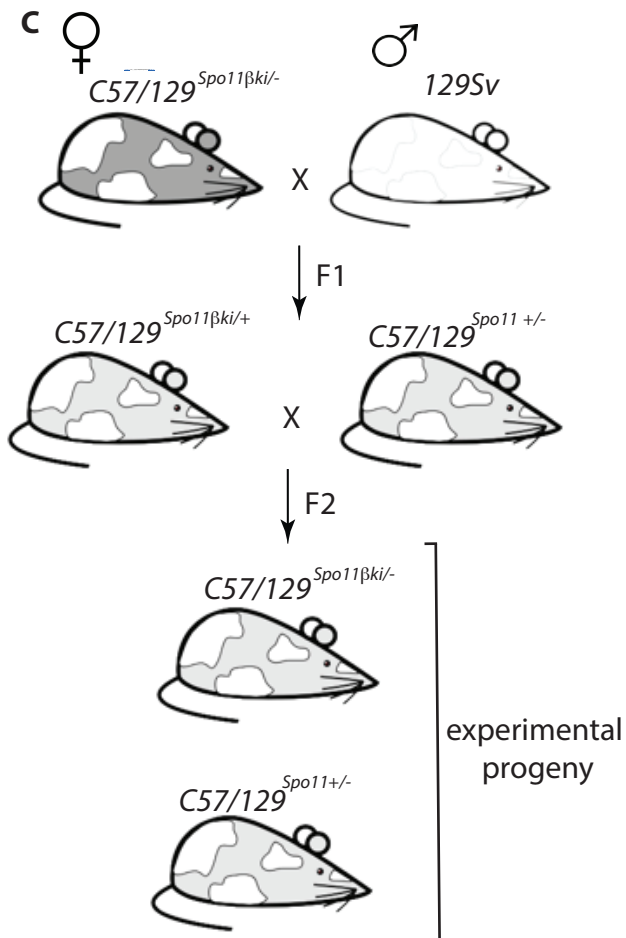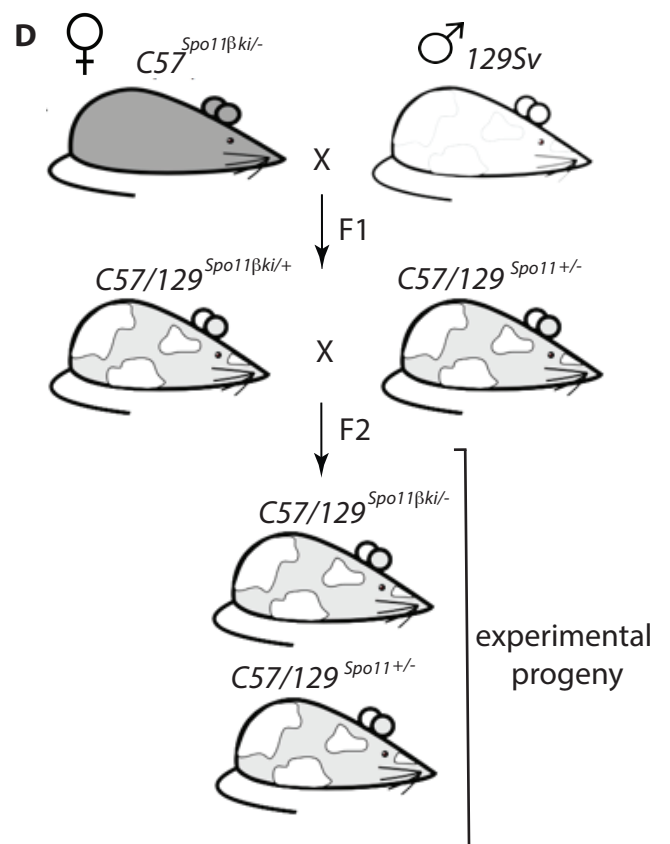

Supplement: Supplementary file 2 — Supplementary file2 (PDF 85 KB) [file 18_2023_4912_MOESM2_ESM.pdf]

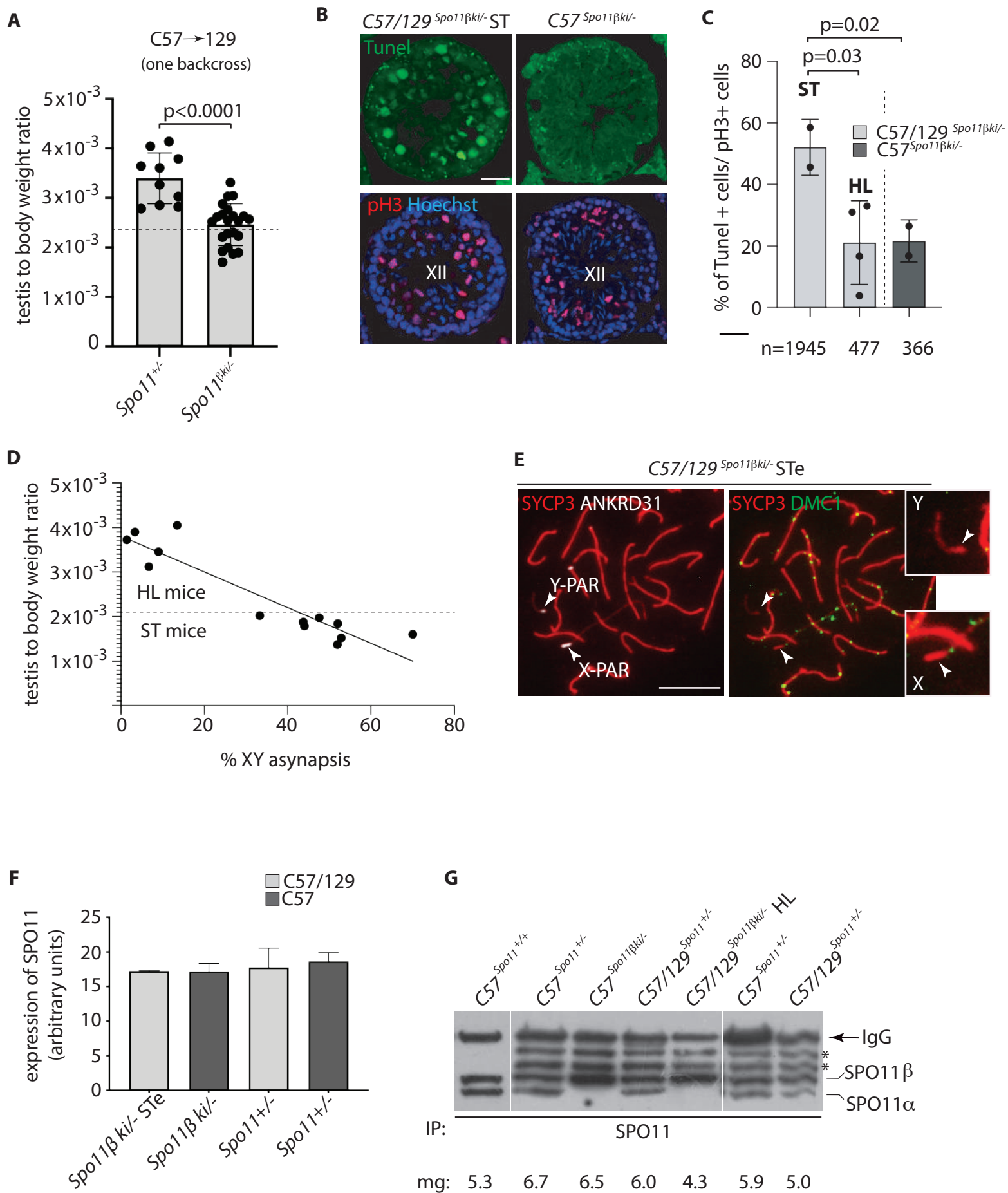

Fig. S3 related to Fig. 1 and Fig. 2

Supplement: Supplementary file 3 — Supplementary file3 (PDF 200 KB) [file 18_2023_4912_MOESM3_ESM.pdf]

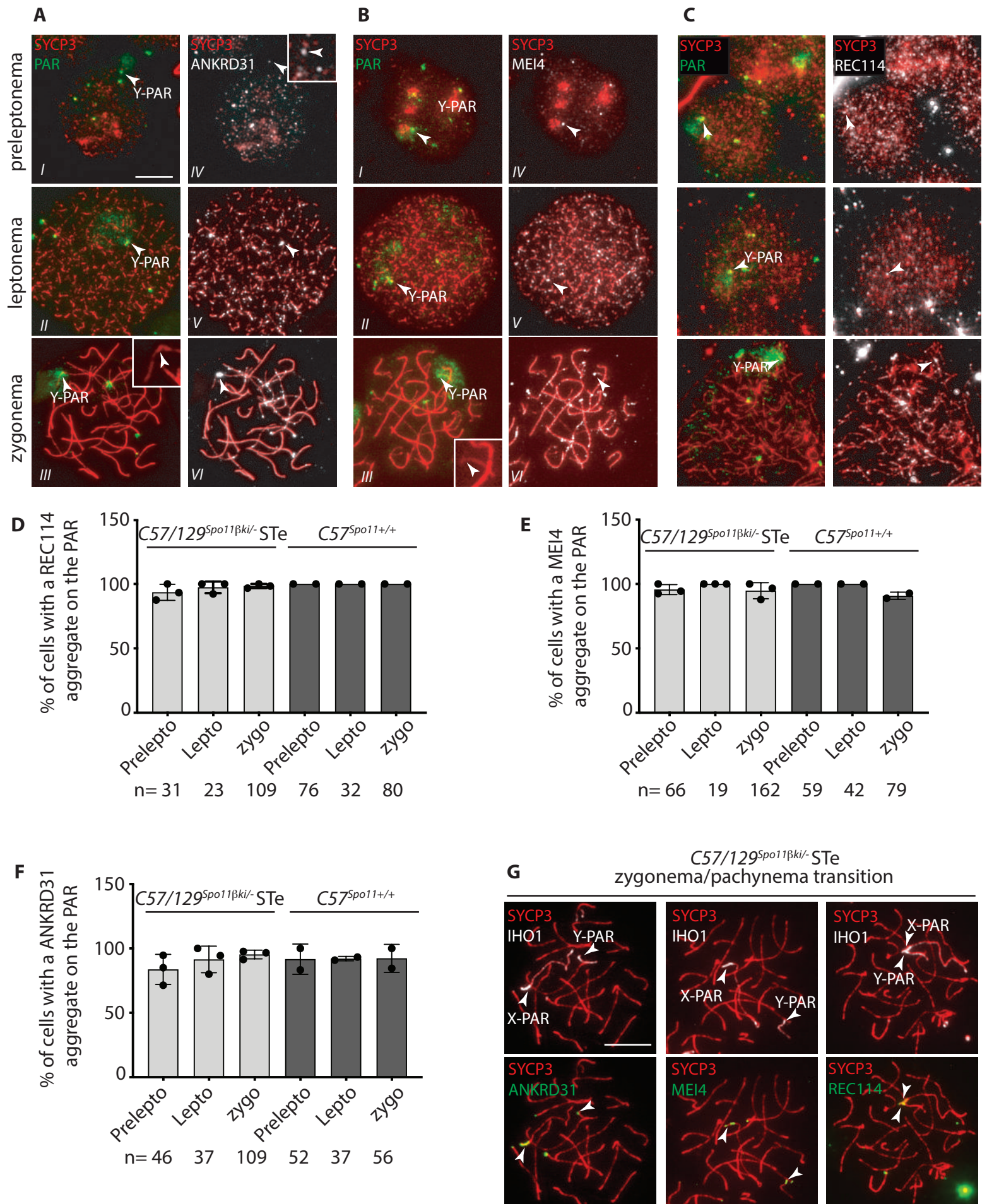

Fig. S4

Supplement: Supplementary file 4 — Supplementary file4 (PDF 630 KB) [file 18_2023_4912_MOESM4_ESM.pdf]

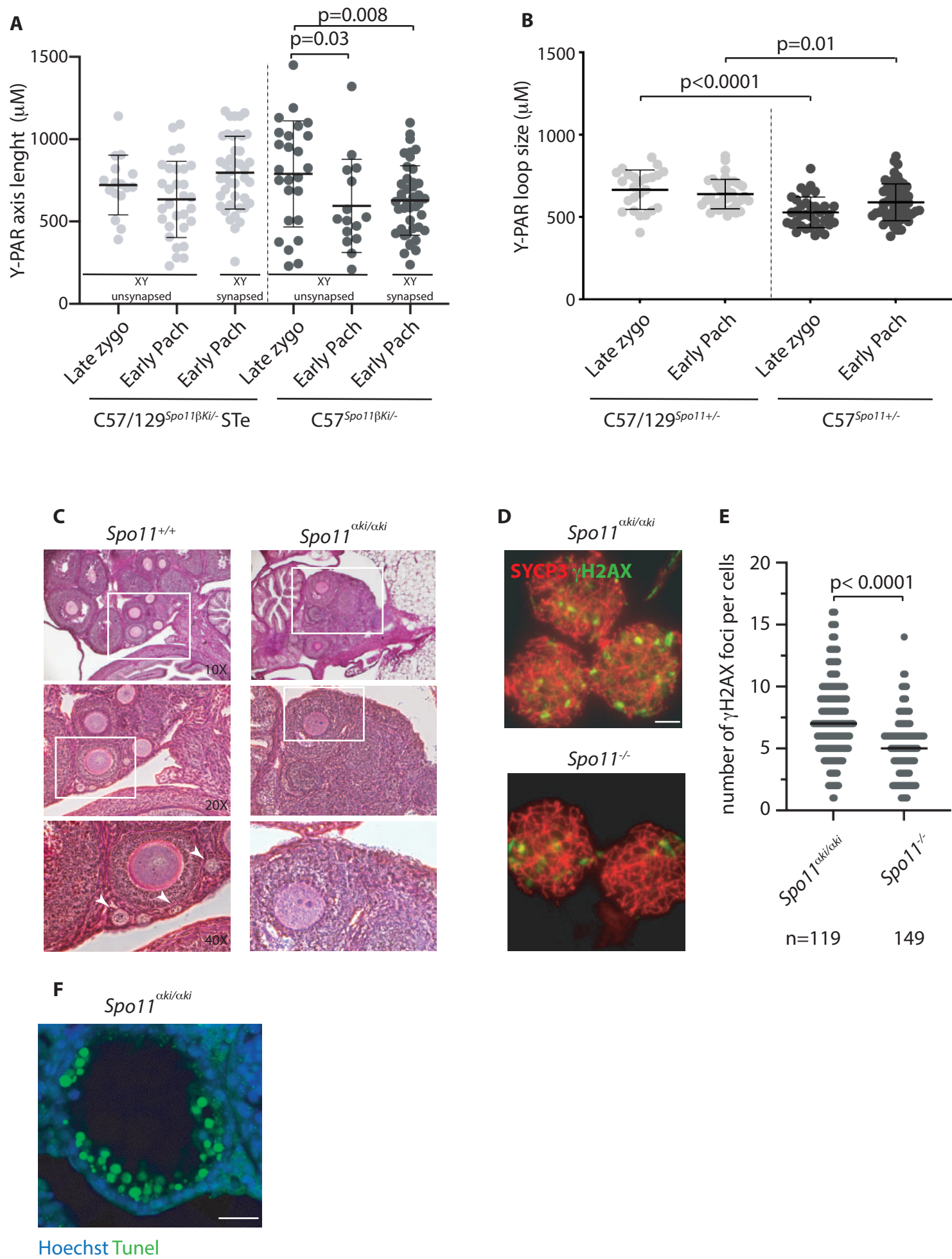

Fig. S5 related to Fig. 3

Supplement: Supplementary file 5 — Supplementary file5 (PDF 298 KB) [file 18_2023_4912_MOESM5_ESM.pdf]

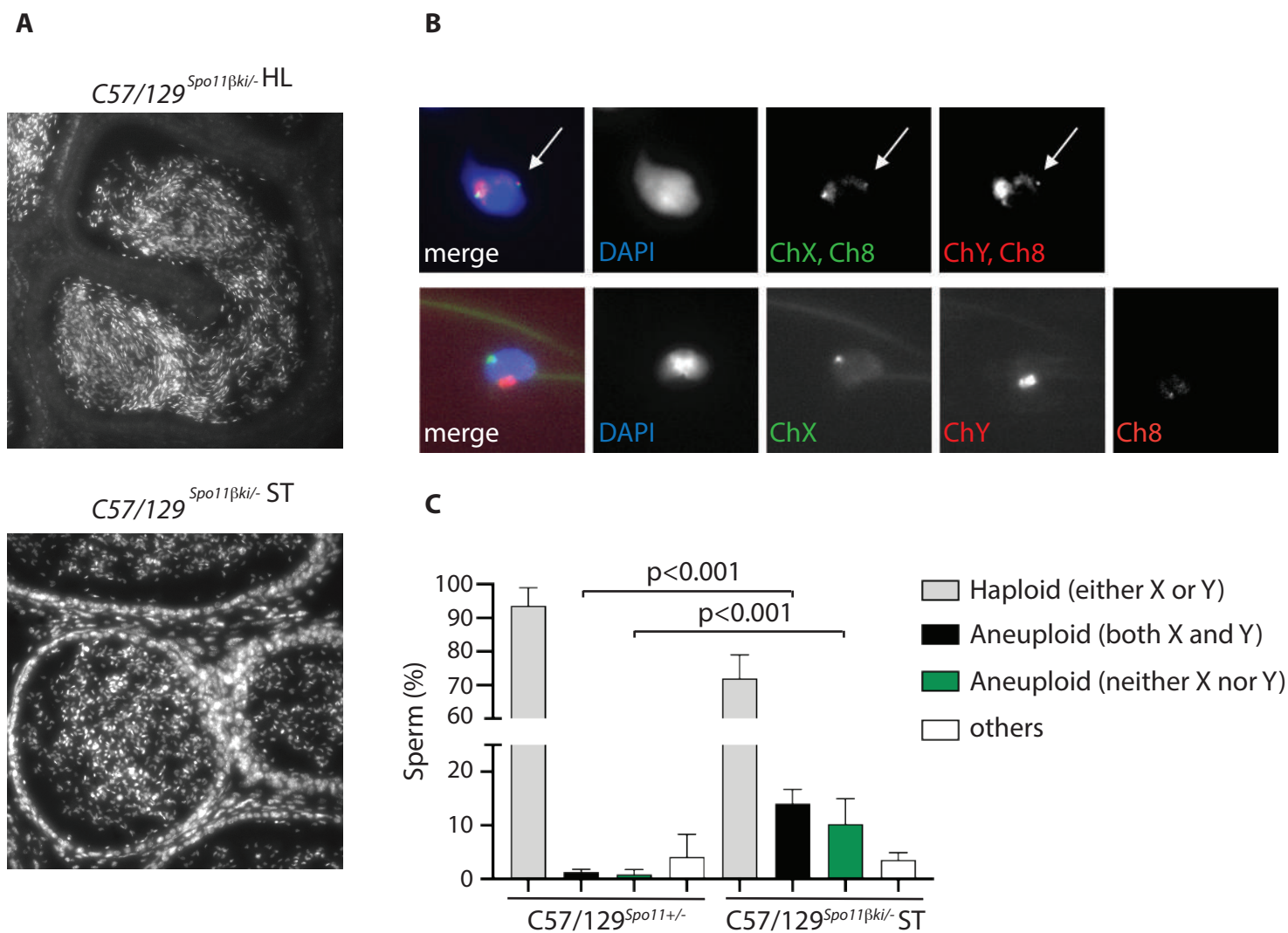

Fig. S6

Supplement: Supplementary file 6 — Supplementary file6 (PDF 7402 KB) [file 18_2023_4912_MOESM6_ESM.pdf]
