# Supplementary material for: The proper interplay between the expression of Spo11 splice isoforms and the structure of the pseudoautosomal region promotes XY chromosomes recombination
Source: Cell Mol Life Sci. 2023 Sep 8;80(10):279. doi: 10.1007/s00018-023-04912-7 (PMC10491539; doi:10.1007/s00018-023-04912-7)
Supplement: Supplementary file 7 — Supplementary file7 (DOCX 32 KB) [file 18_2023_4912_MOESM7_ESM.docx]

**Supplementary Figures Legends and Tables**

**Fig. S1. Targeting of *Spo11βb* and *Spo11αb* cDNA.** A) Schematic of the mouse *Spo11* locus and targeting construct (pDTA-*Spo11*) used for destroying endogenous Exon-1. The black boxes represent exons. Keo is the neomycin resistant cassette, flanked by two LoxP sites (LKL). TA is the targeted allele containing the LKL cassette. The *Spo11Ki* allele was obtained by removing the LKL cassette. Hi is the hybrid intron, while pA is the polyadenylation signal. The position of the 5’ probe used for Southern blotting screening of cells and mice carrying TA is shown. B) Southern blot analysis of genomic DNA from mice carrying the TA *Spo11βKi* allele, before (TA) and after (*Spo11βKi*) LKL cassette removal.

**Fig. S2. Breeding strategies.** A) Crosses made to obtain C57/*129^Spo11βki/-^* and C57/*129^Spo11+/-^* siblings, from either male or female C57/*129^Spo11βki/+^* founders. B) Backcross of (either male or female) C57/*129^Spo11βki/+^* mice in C57. C57/*129^Spo11βki/+^* mice were crossed with C57 wild-type mice to obtain F1 C57/*129^Spo11βki/+^* mice (first backcross). Next, C57/*129^Spo11βki/+^* (either male or female) mice of the F1 were again crossed with a wild type C57 mouse (second backcross). This breeding scheme was executed a total of seven times. C) Backcross of C57/*129^Spo11βki/-^* mice in 129Sv. C57/*129^Spo11βki/-^* females were crossed with a 129Sv wild-type male to obtain a C57/*129^Spo11βki/+^* and C57/*129^Spo11+/-^* progeny. Next, mice of F1were crossed with each other to obtain F2 C57/*129^Spo11βki/-^* and C57/*129^Spo11+/-^* siblings. D) Backcross of C57*^Spo11βki/-^* mice in 129Sv. C57*^Spo11βki/-^* females were crossed with a 129Sv wild type male to obtain the F1 C57/*129^Spo11βki/+^* and C57/*129^Spo11+/-^* progeny. Next, the latter were crossed with each other to obtain F2 C57/*129^Spo11βki/-^* and C57/*129^Spo11+/-^* siblings. In the figure, mice with uniform light grey colour are pure 129 background, while those with dark-grey uniform colour are pure C57Bl/6 background. Mice with a spotted white and grey coat are mixed genetic background. Mice with light spotted coat are those backcrossed once in 129.

**Fig. S3.** A) Relative testicular weight in mice of the indicated genotypes, after one backcross of mice with a pure genetic C57 background in 129 background. Each dot on the graph represents a mouse. B) Apoptosis of metaphase I cells by the TUNEL assay, in mice with the indicated genotypes. Metaphase-I cells at stage XII of the epithelial cell cycle were identified by staining sections with the anti phospho-histone H3 antibody (pH3). Hoechst was used to identify cell nuclei. Magnification bar is 50 μm. C) Quantification of apoptosis of MI cells in mice with the indicated genotypes and genetic backgrounds. Each dot represents a mouse; n is the total number of cells analyzed. Error bars are mean ± standard deviation (SD) of the mean; p indicates statistical significance (p<0.05), one-tailed t-test. D) Linear regression between weight to body ratio and frequency of XY asynapsis in adult C57/129 *^Spo11βki/-^* mice. The dotted line indicates the average weight to body ratio value set in Fig. 1A. Testes with a weight to body ratio above the dotted line are HL, while those below are ST. E) Representative images of surface chromosome spreads of juvenile C57/129 *^Spo11βki/-^* STe mice stained with the indicated antibodies. The white arrows point to the Y and X PARs marked by ANKRD31. Magnifications show the absence of DMC1 foci in PARs. The analyses were carried out on three animals. Magnification bar is 10 μm. F) Densitometric analysis of SPO11/IP western blot from mice with the indicated genotype and genetic background. Each bar is the average expression of seven testes of different mice with the same genotype (two independent experiments). Error bars are mean ± standard deviation (SD) of the mean. G) IP Western blot analysis of SPO11 expression in adult mice with the indicated genotypes and backgrounds. Asterisks mark lower mobility bands that are likely originating from the *Spo11* knockout allele in the model in our supply, expressed in more advanced cell types. [1-3]. Mg= total immunoprecipitated protein per testis (equivalent to one testis per lane). IgG= immunoglobulin.

**Fig. S4.** Representative images of spermatocytes spread chromosomes, of juvenile C57/*129^Spo11βki/-^* STe mice. Cells were stained with SYCP3 and with the PAR FISH probe, in combination with anti-ANKRD31 (A), MEI4 (B), or REC114 (C) antibodies. Frequency of the presence of aggregates of REC1141 (D), MEI4 (E), and ANKRD31 (F) on PAR, in mice with the indicated genotypes and backgrounds. n= number of cells analyzed. G) Representative images of spermatocytes spread chromosomes at the zygotene-pachytene transition, from C57/*^129Spo11βki/-^*mice. The IHO1 staining pattern was used to identify zygote-pachytene transition stage cells and PAR. Cells were co-stained with SYCP3, in combination with ANKRD31, MEI4 or REC114. Magnification bars in A and G are 10μm.

**Fig. S5.** A) Measurements of PAR-axis length from conventional immune-FISH images of cells at late zygonema and early pachynema, in mice with the indicated genotypes and backgrounds. Each dot represents measurements of a single cell. Three mice analyzed per genotype. B) Measurements of loop-axis extension from conventional immune-FISH images of cells at late zygonema and early pachynema, in mice with the indicated genotypes; two mice analyzed per genotype. Each dot represents measurements of a single cell. C) Representative images of ovaries sections stained with Hematoxylin and periodic acid Schiff, in mice with the indicated genotypes. The white arrows point to the primordial follicles. D) Surface chromosome spreads of mice with the indicated genotypes, stained with the anti-SYCP3 and γH2AX antibodies. Magnification bar is 10μm. E) quantification of the number of γH2AX patches in cells of mice with the indicated genotypes; p indicates statistical significance (p<0.05), two-tailed t-test. n= number of cells analyzed. F) Representative image of TUNEL positive cells (green) in seminiferous tubules of mice expressing the single SPO11α splicing isoform. Hoechst stains cells nuclei. Magnification bars is 50μm.

**Fig. S6**. A) Representative images of the cauda of the epididymis of mice with the indicated genotypes and genetic background, stained with Hoechst. B) Top panel, representative image of mouse sperm from C57/129*^Spo11βki/-^* ST mice, stained with FISH probes against ChX (green), ChY (red) and Ch8 (green and red). The arrows point Ch8. In the bottom panel Ch8 was stained with Alexa Fluor-647. C) Quantification sperms haploid and aneuploid for the sex chromosomes, in mice with the indicated genotypes (C57/129*^Spo11+/-^* n= 802; C57/129*^Spo11βki/-^* ST n=293). In the graph, “others” indicate aneuploidy cases found with low frequency (Ch8 and ChY diploidy). p indicates statistical significance (p<0.05), error bars are SE, chi-squared test.

References:

1. Kauppi L, Barchi M, Baudat F, Romanienko PJ, Keeney S, Jasin M. Distinct properties of the XY pseudoautosomal region crucial for male meiosis. Science. 2011;331(6019):916-20. Epub 2011/02/19. doi: 10.1126/science.1195774. PubMed PMID: 21330546; PubMed Central PMCID: PMCPMC3151169.

2. Kauppi L, Barchi M, Lange J, Baudat F, Jasin M, Keeney S. Numerical constraints and feedback control of double-strand breaks in mouse meiosis. Genes Dev. 2013;27(8):873-86. doi: 10.1101/gad.213652.113. PubMed PMID: 23599345; PubMed Central PMCID: PMCPMC3650225.

3. Lange J, Pan J, Cole F, Thelen MP, Jasin M, Keeney S. ATM controls meiotic double-strand-break formation. Nature. 2011;479(7372):237-40. Epub 2011/10/18. doi: 10.1038/nature10508. PubMed PMID: 22002603; PubMed Central PMCID: PMCPMC3213282.
